# Sagittal spinopelvic malalignment in degenerative scoliosis patients: isolated correction of symptomatic levels and clinical decision-making

**DOI:** 10.1186/s13013-018-0174-y

**Published:** 2018-12-27

**Authors:** Steven M. Presciutti, Philip K. Louie, Jannat M. Khan, Bryce A. Basques, Comron Saifi, Christopher J. Dewald, Dino Samartzis, Howard S. An

**Affiliations:** 1 Department of Orthopaedics, Emory University, Atlanta, GA USA; 2 Department of Orthopaedic Surgery, Rush University Medical Center, 1611 W. Harrison St., Suite 300, Chicago, IL 60612 USA; 3 Department of Orthopaedic Surgery, University of Pennsylvania, Philadelphia, PA USA

**Keywords:** Sagittal imbalance, Fractional curve, Degenerative scoliosis, Spinal fusion, Symptomatic levels

## Abstract

**Background:**

This study aims to determine if (1) loss of lumbar lordosis (LL), often associated with degenerative scoliosis (DS), is structural or rather largely due to positional factors secondary to spinal stenosis; (2) only addressing the symptomatic levels with a decompression and posterolateral fusion in carefully selected patients will result in improvement of sagittal malalignment; and (3) degree of sagittal plane correction achieved with such a local fusion could be predicted by routine pre-operative imaging.

**Methods:**

A retrospective study design with prospectively collected imaging data of a consecutive series of surgically treated DS patients who underwent decompression and instrumented fusion at only symptomatic levels was performed. Pre- and post-operative plain radiographs and pre-operative magnetic resonance imaging (MRIs) of the spinopelvic region were analyzed. LL, pelvic incidence (PI), pelvic tilt (PT), and sacral slope (SS) were assessed in all patients. As a requirement for the surgical strategy, all patients presented with a pre-operative PI-LL mismatch greater than 10^°^. Post-operative complications were assessed.

**Results:**

Pre-operative MRIs and lumbar extension radiographs revealed a mean LL of 42^°^ (range 10–66^°^) and 48^°^ (range 20–74^°^), respectively, in 68 patients (mean follow-up 29 months). LL post-operatively was corrected to a mean PI-LL of 10^°^. Of patients who achieved PI-LL mismatch within 10^o^ on their pre-operative extension lateral lumbar radiographs, 62.5% were able to maintain a PI-LL mismatch within 10^°^ on their initial post-operative films. Only 37.5% were not able to achieve that mismatch on extension radiographs (*p* = 0.001, OR = 9.58). Similarly, 54.2% were able to achieve a PI-LL < 10^°^ on initial post-operative radiographs, when pre-operative MRI revealed a PI-LL mismatch within 10^°^. In contrast, only 20.5% achieved that goal post-operatively if their mismatch was greater than 10^o^ on their MRI (*p* = 0.003, OR = 4.25).

**Conclusion:**

With a decompression and instrumented fusion of only the symptomatic levels in symptomatic DS patients, we were able to achieve a PI-LL mismatch to within 10^°^. The loss of LL observed pre-operatively may be largely positional rather than structural. The amount of LL correction observed immediately after surgery can be predicted from pre-operative lumbar extension radiographs and supine sagittal MRI.

## Introduction

With an aging population and the growing ability to manage difficult health problems in later years, the effective management of adult degenerative scoliosis (DS) is more critical than ever. The prevalence of DS ranges widely in the literature from 8.3 to 68% of the adult population, with a higher prevalence in older patients [1, 2]. DS is a complex spine presentation that commonly involves a combination of both degenerative spinal stenosis and deformity. In addition, DS usually presents with foraminal stenosis, most often on the concave side of the fractional lumbosacral curve (the compensatory curve at the level of the lumbosacral junction below the major curve) and less often with central canal stenosis [3–6]. Optimizing the treatment for this pathology is similarly multifaceted with several treatment algorithms that are widely practiced and described in the literature. The treating surgeon must balance the reward of providing clinical benefit and radiographic improvement with the measurable risks and expense incurred with these surgical interventions.

The optimal method to address mild-moderate sagittal imbalance in DS patients remains an ongoing debate. When non-operative treatment fails to effectively manage a patient’s symptoms, several surgical options are available [3, 7–11]. An instrumented posterior spinal arthrodesis is often performed in combination with decompression of the symptomatic levels [9–12]. The options for choosing which levels to fuse include (1) fusing only those levels that are symptomatic stenotic levels (local), (2) fusing the majority of the levels that comprise the DS curve (regional), or, in specific cases, (3) extend the fusion to the upper thoracic spine with or without associated osteotomies (global). Compared to a local fusion surgery, global arthrodesis procedures with osteotomies may provide the greatest potential for sagittal radiographic improvement in fixed deformities. However, these larger surgeries are associated with several additional risks: proximal junctional kyphosis (PJK), elevated perioperative blood loss, longer operative times, and neurologic complications [11, 13, 14]. Regardless, the goal of the procedure should be to achieve an adequate decompression of the neurologically symptomatic levels. Traditionally, a major goal of surgery has been to correct the LL to within 10^o^ of the PI for improved health-related quality of life scores post-operatively [15–18]. However, new research has indicated that the age of the patient should be taken into account when determining the correction to the “ideal” sagittal alignment, as older patients requiring less rigorous alignment objectives [18].

Loss of LL is commonly observed in patients with symptomatic DS. A critical distinction for the surgeon to make with regard to which levels to fuse revolves around the etiology of the loss of LL and resulting positive sagittal imbalance commonly observed in these patients. The surgeon must decide whether this loss of LL is largely structural or whether it is more positional in nature given that stenosis is usually present in this setting. This distinction specifically affects the decision regarding whether or not the scoliotic curve needs to be included in the fusion (regional or global fusion) or if a locally targeted fusion of only the symptomatic stenotic levels can be performed. As such, if the loss of LL is positional, the question remains whether spinal flexibility as assessed on supine or extension-based pre-operative imaging can help predict the amount of sagittal plane correction that can be obtained by addressing only the symptomatic stenotic levels.

The aims of our study were multifactorial. First, we explored whether the loss of LL in a consecutive series of DS patients was structural or rather largely due to positional factors secondary to spinal stenosis. Secondly, we determined if a targeted decompression and instrumented fusion of only the symptomatic stenotic levels in DS patients with mild-moderate sagittal imbalance led to a significant radiographic improvement in sagittal profile. Finally, we determined if the degree of sagittal plane correction achievable with this technique could be predicted by routine pre-operative imaging, such as standing extension lumbar radiographs and supine lumbar magnetic resonance imaging (MRI).

## Methods

A retrospective design with prospective imaging data collection study was performed on a consecutive series of DS patients operated by a single surgeon. Following institutional review board (IRB) approval, we obtained radiographic and clinical data on 114 patients diagnosed with DS between February 2006 and September 2014. Inclusion criteria entailed patients who failed conservative treatment for DS, presented with a loss of normal LL as observed on pre-operative radiographs, “moderate” scoliosis with a Cobb angle < 40^o^, coronal shift less than 2 cm, and a sagittal vertical axis (SVA) less than 10 cm, and who underwent a decompression and instrumented posterolateral inter-transverse process arthrodesis of only their neurologically symptomatic level(s). Every patient failed conservative therapies (i.e., anti-inflammatory medications, physical therapy, and/or epidural steroid injections) before undergoing surgery. We excluded patients if they had undergone a previous fusion, concomitant osteotomy procedure, or had less than 12 months of follow-up. Of the original 114 patients identified, 68 fulfilled the above criteria. Patient demographics, intraoperative complications, and re-operation data were collected on all patients. All patients had pre- and post-operative full-length standing scoliosis radiographs in which they were instructed to keep their knees extended and their hands on their clavicles. In addition, pre-operative standing lumbar flexion and extension radiographs as well as pre-operative supine sagittal MRIs were available for all patients. Plain radiographic and MRI measurements were made using the Opal-RAD Digital Radiology Suite® (Konica Minolta Medical Imaging, Garner, NC).

**Fig. 1:**
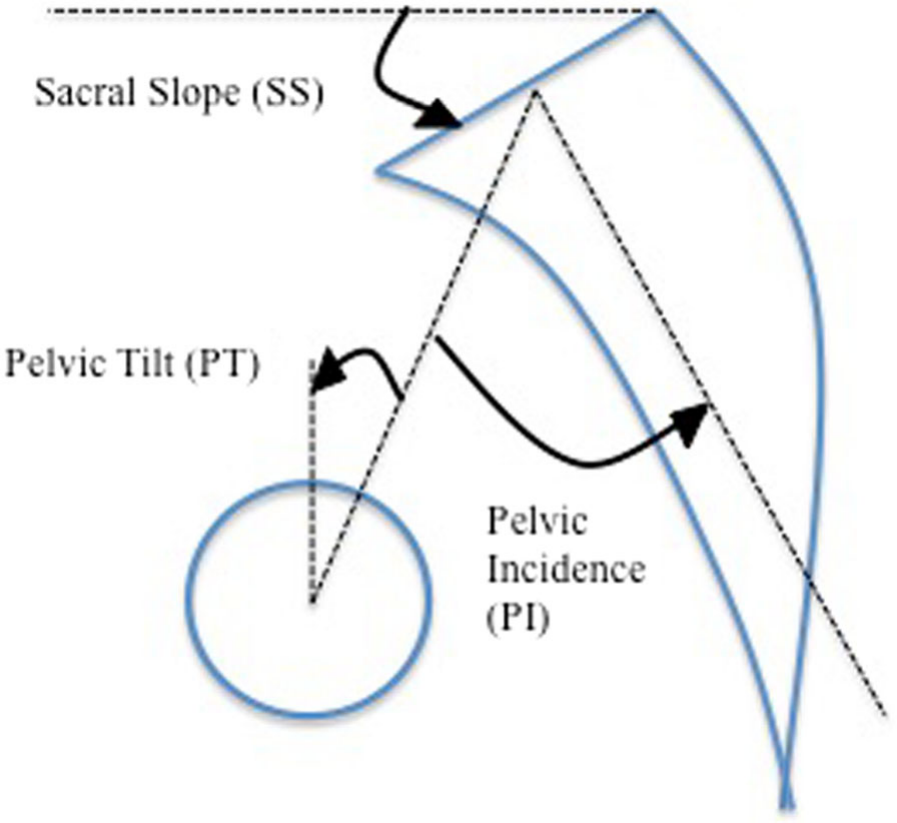
The pelvic incidence (PI) is the angle between the line perpendicular to the midpoint of the superior sacral endplate and a line through the midpoint of the superior sacral endplate to the center of the femoral heads. Pelvic tilt (PT) is the angle between a line drawn from the center of the S1 endplate to the center of the femoral head and a second vertical reference line intersecting the center of the femoral head. Sacral slope (SS) is the angle between a line drawn parallel to the S1 endplate and a second horizontal reference line

Lumbar lordosis, described as the resultant angle of the L1 superior endplate and the S1 superior endplate, was measured on all imaging, including the supine MRIs. Furthermore, foraminal height was measured on lateral pre-operative radiographs as the largest measured distance between the superior margin of the inferior vertebral pedicle and inferior margin of the superior vertebral. The reported foraminal height was taken from the level that showed the smallest foraminal dimension within the neurologically symptomatic segments. The remaining parameters were analyzed on pre- and post-operative full-length scoliosis radiographs only (Fig. 1). PI was measured as the resultant angle of a line through the midpoint of the superior sacral endplate to the center of the femoral heads and the line perpendicular to the midpoint of the superior sacral endplate. Sacral slope (SS) was defined as the angle between the line through the superior sacral endplate and a horizontal reference line. Lastly, pelvic tilt (PT) was noted as the resultant angle between a line through the midpoint of the superior sacral endplate to the center of the femoral heads and a vertical reference line.

All data were collected and entered into a spreadsheet. Descriptive statistics was performed of the variables, noting mean, range, and standard deviation (±) where applicable. Three independent members of the research team performed all measurements (plain radiographs and MRI). Inter-rater reliability was calculated using a Pearson coefficient test and a strong correlation was considered if *r* > 0.80 [19]. The three sets of measurements were then averaged for final statistical analysis. Continuous data was compared using a student’s *t* test; dichotomous data was compared using Fisher’s exact test and an odds ratio test. A receiver operating characteristic (ROC) analysis was performed in order to directly compare the ability of standing lumbar extension radiographs and supine MRIs to predict successfully maintained PI to LL mismatch of 10^o^ or less at a mean of 29 months. The area under the ROC curve (AUC) was subsequently calculated where a value of 1 represents a perfect fit. Based on the AUC, the accuracy of the imaging studies to predict lumbar lordosis correction was classified as the following: 0.9–1.0 (excellent), 0.8–0.9 (good), 0.7–0.8 (fair), 0.6–0.7 (poor), and 0.5–0.6 (fail) [20]. All statistical tests were performed using PASW statistics software for Windows, version 18.0 (IBM, Armonk, NY). The threshold for statistical significance was established at *p* < 0.05; 95% confidence intervals (CI) were also noted to assess the precision and significance of the associations.

## Results

**Fig. 2:**
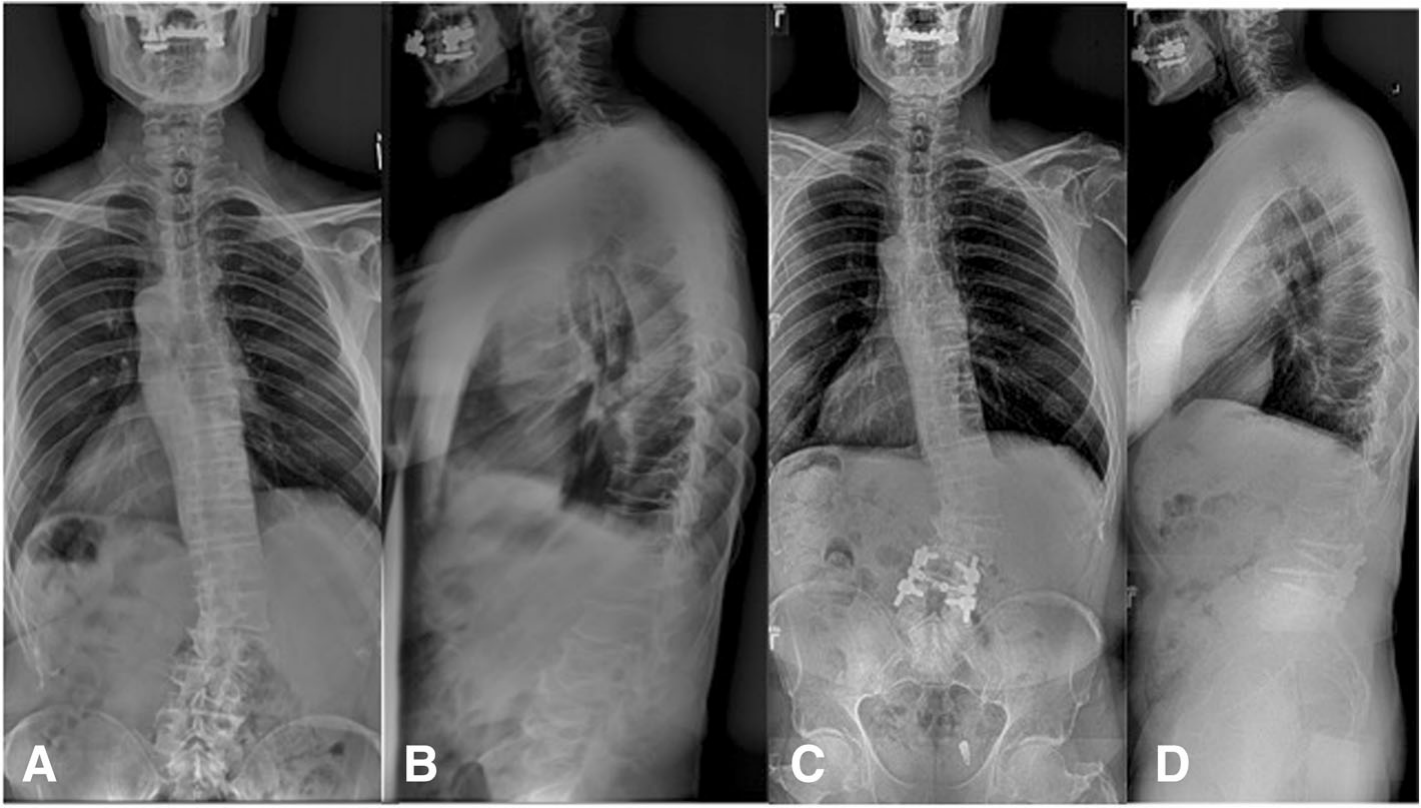
Anteroposterior (AP) (**a**) and lateral (**b**) full-length spine radiographs of a 63-year-old male that presented with mild axial back pain and progressively worsening L4 radicular symptoms down the right lower extremity. After failing conservative measures, the goal of the surgery was to simply address the symptomatic levels. There was no significant deformity or instability. Post-operative AP (**c**) and lateral (**d**) full-length spine radiographs showing the laminectomy, foraminotomy, and posterolateral fusion that was performed at L4-5

Radiographic and clinical data were collected from the 68 consecutive patients that met the inclusion and exclusion criteria (Table 1). The mean age of the 24 males and 44 females was 68 ± 8.3 years (range 42–86 years). The mean follow-up was 29.2 ± 19.1 months (range 12–108 months). All patients presented with radicular symptoms with varying amounts of low back pain. Most commonly, the instrumented arthrodesis was performed from L4 to S1 (*n* = 17) followed by L3-S1 (*n* = 12) (Fig. 2).

**Table 1:** Demographics for the final cohort of included patients

Parameter	Cohort (*n* = 68)
Age (years)	68.1 ± 8.3
Gender	
Male	24 (35%)
Female	45 (65%)
Follow-up (months)	29.2 ± 19.1
Prior decompression	12 (17%)

**Fig. 3:**
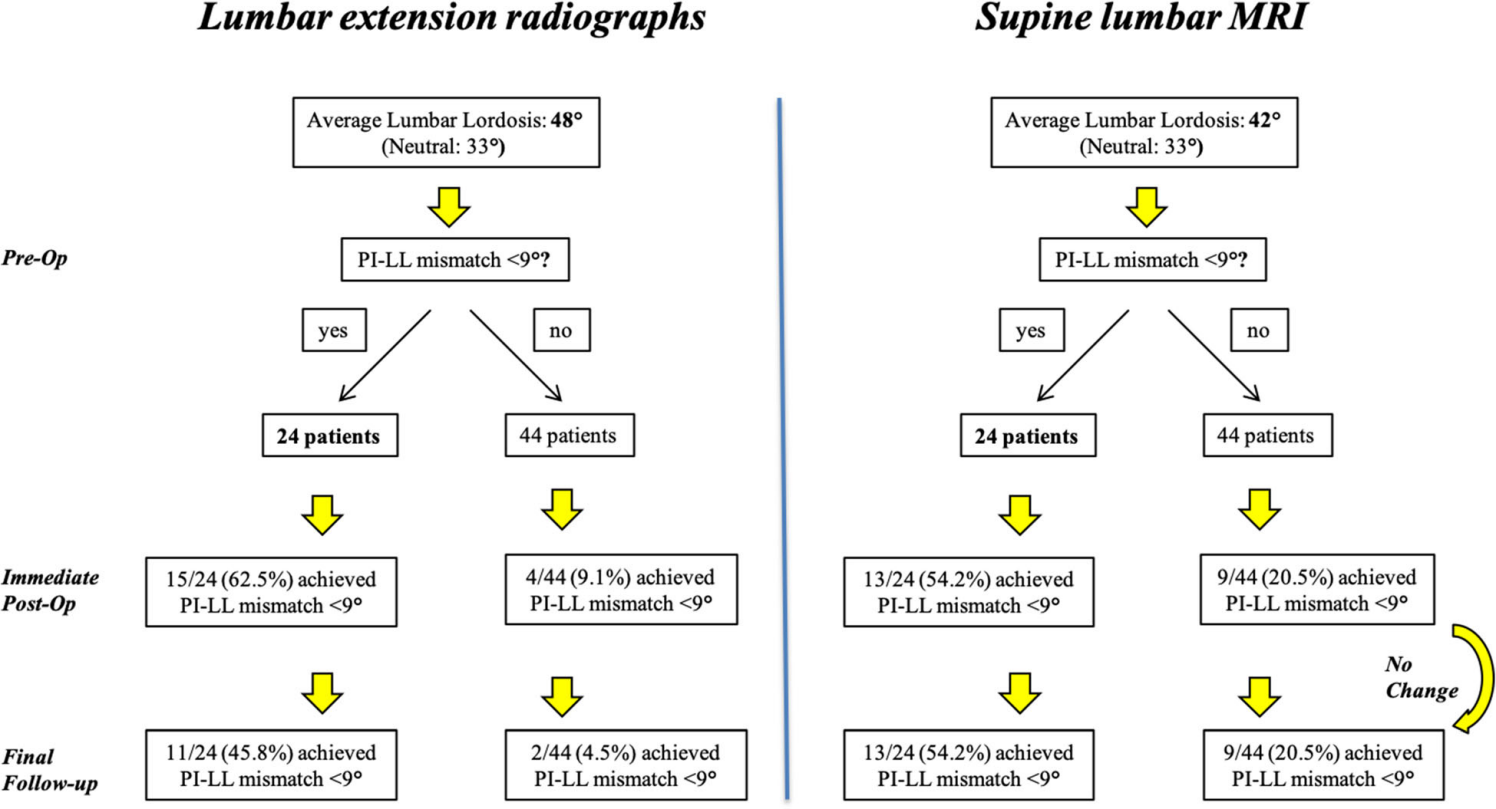
Flowcharts illustrating the ability of pre-op lumbar extension radiographs and supine MRIs to predict an achievable radiographic correction. These findings support the notion that the hypo-lordosis seen in DS may be largely positional and compensatory to the associated spinal stenosis in these patients

All radiographic reliability values showed excellent agreement with *r* values above 0.95 and *p* values < 0.0001. The mean pre-operative and post-operative PI was 53^o^ (± 13.0^o^) and 53.8^o^ (± 11.3^o^), respectively (Table 2). The mean pre-operative LL was 32.6° (± 14.5^o^) while in a standing position of comfort while the mean LL on pre-operative standing extension films was 48° (± 12.1^o^) (Fig. 3). Similarly, the mean LL on pre-operative supine MRIs was 42° (± 11.4^o^). The mean LL difference between the pre-operative MRI and the pre-operative standing lateral spine radiograph was 9.6° (*p* < 0.001). Likewise, this difference was 16^o^ (*p* < 0.001) between pre-operative extension and neutral lumbar radiographs, indicating just how flexible these curves can be. The LL on their first post-operative standing radiographs (mean 2.5 weeks) was found to be corrected to a mean of 43.6° (range 14 to 67°). Therefore, the mean amount of LL correction achieved was 11° (*p* < 0.001). After a mean follow-up of 29.2 months, however, some of the initial LL correction was lost with a mean of 39° (*p* < 0.001). In addition, the mean foraminal height within the fused segments was 15.4 mm (± 2.7 mm). These results, along with the values for SS and PT, are summarized in Table 2.

**Table 2:** Measured radiographic parameters

Radiographic parameter	Mean (degrees) ± SD	Range	*p* value
Lumbar lordosis			
Pre-op	32.6 ± 14.5	[2.4–60.0]	
Immediately post-op	43.6 ± 11.6	[14.0–67.9]	< 0.001
Final follow-up	39.0 ± 12.1	[9.1–64.4]	< 0.001
Pelvic incidence			
Pre-op	53.0 ± 13.0	[25.3–81]	
Immediately post-op	53.8 ± 11.3	[30.4–83.2]	0.902
Sacral slope			
Pre-op	28.2 ± 10.8	[5.6–60.4]	
Immediately post-op	26.9 ± 10.0	[3.7–57.2]	0.125
Pelvic tilt			
Pre-op	23.9 ± 9.5	[2.2–45.7]	
Immediately post-op	27.0 ± 9.5	[10.2–46.6]	0.073
Foraminal height (mm)	15.4 ± 2.7	[9.1–25.1]	

*SD* standard deviation

Taking into account the post-operative LL as it relates to PI, decompression and local instrumented fusion of only the neurologically symptomatic levels in our cohort of DS patients resulted in a post-operative PI-LL mismatch of 10°. The mean pre-operative PI-LL difference was 19^o^, and this correction after surgery was found to be significant (*p* < 0.001). As noted above, some of this correction was lost at the final follow-up at a mean of 29.2 months and the PI-LL mismatch increased to 14.8^o^. This was nonetheless still a significant improvement (*p* < 0.001). Our cohort did have a small increase in PT post-operatively. That being said, it is hard to know how to interpret this given that the increase was not statistically significant. It is important to note, however, that pelvic retroversion is often found to be a compensatory mechanism in patients with a positive sagittal imbalance that allows them to maintain an upright posture. The goal of surgery should always be to decrease PT in sagittally imbalanced patients, particularly since it has been shown that patients with a larger post-operative PT, increased pelvic retroversion, were more likely to demonstrate residual pain, than patients with a smaller post-operative PT [21].

**Fig. 4:**
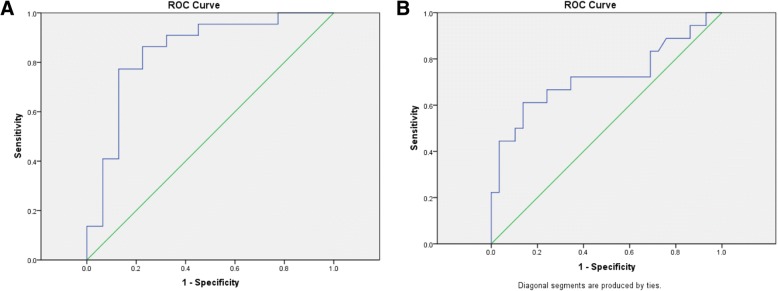
Receiver operating curves for utilizing pre-operative extension lumbar spine radiographs (**a**) and pre-operative supine sagittal MRI (**b**) as a predictor of sagittal balance correction (PI-LL mismatch ≤ 10^o^)

In order to try to determine which patients were more susceptible to this gradual loss of LL over time, we compared the LL found on their pre-operative lateral extension radiographs and supine MRI to the LL found on their pre-operative standing spinal radiographs to investigate whether curve flexibility pre-operatively could be used to predict the behavior of the curve post-operatively. The hypothesis was that if a patient’s curve was less structural to begin with, then, it may be less prone to lose lordosis over time, and that this would be predictive based on the commonly acquired pre-operative imaging. Interestingly, patients who were able to correct their PI-LL mismatch to 10^o^ or less on their pre-operative lumbar extension radiographs had a significantly better chance of achieving and maintaining that goal post-operatively (Fig. 4). Out of 24 patients who were able to achieve that mismatch on extension radiographs, 15 (62.5%) of them were found to be within 10^o^ on their immediate post-operative films. Alternatively, if patients were not able to achieve a PI-LL mismatch of 10^o^ or less on their pre-operative radiographs, only 4 patients (14.8%) were found to have it post-operatively. This difference was statistically significant (*p* = 0.001, OR = 9.58). This same result was maintained even at the final follow-up. This is in contrast to only 9.1% of patients that were able to achieve this mismatch post-operatively without being able to achieve their pre-operative standing extension radiographs (*p* = 0.006, OR = 6.77, 95% CI 1.60–28.69).

Similar results were found with regard to the amount of LL present on their pre-operative supine lumbar MRIs. We found that patients who were able to correct their PI-LL mismatch to 10^o^ or less on their MRI had a significantly better chance of achieving that goal post-operatively, regardless of what their PI-LL mismatch was on standing lumbar radiographs (pre-op 35.3%; immediate post-op 62.5%; final 45.8%). In fact, out of 24 patients who were able to achieve that mismatch of 10^o^ or less on pre-operative supine MRI, 15 (62.3%) of them were found to have their LL within 10^o^ of their PI on their immediate post-operative films. In contrast, only 20.5% of patients achieved that goal post-operatively if their mismatch was greater than 10^o^ on their pre-operative MRI (*p* = 0.003, OR = 4.25, 95% CI 1.19–15.23). This predictive ability of MRI was maintained at final follow-up as well (*p* = 0.003, OR = 4.25, 95% CI 1.19–15.23).

An ROC analysis was performed in order to directly compare the ability of standing lumbar extension radiographs and supine MRIs to predict successfully maintained PI to LL mismatch of 10^o^ or less at a mean of 29 months (Fig. 4). Based on the area under the ROC curve (AUC), lumbar extension radiographs were found to be superior to supine MRIs in predicting this successful sagittal plane correction. Lumbar extension radiographs had an AUC of 0.85 (95% CI 0.73–0.96) while supine MRIs had an AUC of 0.72 (95% CI 0.56–0.89).

In our cohort, there were no intraoperative complications reported. Following the index procedure, however, seven patients (10.3%) did require a revision surgery at a mean of 17.6 months (range 2.2–34.3). Five patients (7.4%) presented with recurrent stenosis at their previously fused segments, all of which were treated with a revision decompression. One patient underwent a decompression and extension of the posterior fusion one level cephalad due to adjacent segment degeneration. One patient experienced prominent instrumentation requiring removal.

## Discussion

As described above, the mean pre-operative LL as measured in our cohort was 32.6° (range 2.4–60°) on neutral lateral lumbar radiographs, which is consistent with previous studies [4, 22]. Our results indicate that we were able to achieve and maintain an adequate reduction of the PI-LL mismatch at a mean of 29.2 months following decompression and local posterior instrumented arthrodesis of only the neurologically symptomatic levels. These results indicate that the hypo-lordosis and positive sagittal balance commonly observed pre-operatively in DS patients is not always entirely structural but rather positional and compensatory for the concomitant spinal stenosis. They also indicate that, in a carefully selected group of patients whose spinal deformity is not yet too big, the PI-LL mismatch can often be reduced close to goal levels without the need for more complex and longer procedures. Moreover, when compared to the LL on routine standing spinal radiographs (taken in the position of comfort), the LL on pre-operative standing lumbar extension radiographs and supine MRIs were found to be significantly greater with mean values of 15^o^ and 9°, respectively. These findings further support the notion that the hypo-lordosis often seen in DS patients may have a positional component.

While many patients with DS do require longer fusion constructs that span across the apex of their curve, we wanted to investigate whether there are select patients in which an appropriate restoration of LL can be achieved by fusing only their neurologically symptomatic levels. These symptomatic levels are typically at the level of the fractional curve, which is caudal to the major DS curve. In the setting of a fractional curve, patients often experience radicular symptoms originating from foraminal stenosis within the fractional curve, rather than from stenosis in the major degenerative curve itself. This important distinction directly affects the decision as to whether or not the entire curve needs to be addressed with longer regional/global fusions, in the case of a structural curve, or whether a lesser targeted decompression and fusion of only the neurologically symptomatic levels can be performed (in the case of a more positional deformity). The importance of distinguishing the etiology of this hypo-lordosis and determining just how structural the curve becomes magnified given the greater rate of complications associated with longer fusion constructs, specifically in this typically older patient population. These higher rates of complications in longer fusions for deformity correction are due to a higher prevalence of PJK, elevated perioperative blood loss, longer operative times, and neurologic injury [11, 13, 14]. As recent awareness regarding the importance of sagittal alignment on post-operative quality of life has come to light, many surgeons may consider the hypo-lordosis in this group of DS patients as the reason to perform longer regional/global instrumented fusions, osteotomies, or interbody fusions in order to bring the PI-LL mismatch to within 10^o^ [15]. Traditionally, a major goal of surgery has been to correct the LL to within 10^o^ of the PI for improved health-related quality of life scores post-operatively [15–18]. However, new research has indicated that the age of the patient should be taken into account when determining the correction to the “ideal” sagittal alignment, as older patients requiring less rigorous alignment objectives [18]. These radiographic parameter targets based on age may provide more “patient-specific” alignment thresholds. In this study, we assessed an overall goal of a PI-LL mismatch to within 10^o^, regardless of the age of the patient. In the future, we plan to conduct a prospective study on this specific cohort, and will adjust target sagittal balance with the age of the patient in consideration.

Multiple studies have investigated this concept of how lumbar hypo-lordosis seen on pre-operative radiographs can change depending on body position. Reduction of LL in the upright position has been shown to be affected by involuntary or pain-related voluntary muscle contraction [23]. Intraoperatively, this effect is diminished by muscle dissection and the muscle relaxation effect of general anesthesia. Harimaya and colleagues analyzed patients with lumbar hypo-lordosis and concluded that those who presented with pre-operative hypo-lordosis, when positioned prone during reconstructive surgery, experienced significantly increased LL compared to their pre-operative standing plain radiographs [24]. Similarly, Peterson et al. showed that positioning a patient prone on an open-frame table increased segmental lordosis by 22% at the L5-S1 level, while also preserving segmental and total lordosis at the remaining levels [25].

To determine if there is a way to predict whether appropriate sagittal plane correction (PI-LL < 10^o^) can be achieved and maintained using this targeted technique in patients with DS and mild-moderate sagittal imbalance, we examined the relationship between the LL present in various pre-operative imaging studies in order to assess spinal flexibility. If a patient was able to achieve a PI-LL mismatch of 10^o^ or less on either their pre-operative extension radiograph or MRI, we found that they were able to achieve that goal mismatch post-operatively 62.5 and 54.2% of the time, respectively. In contrast, if patients were unable to obtain that mismatch pre-operatively, thereby demonstrating a more structural curve, patients achieved a PI-LL mismatch within 10^o^ only 9.1 and 20.5% of the time post-operatively, respectively. Importantly, we report the ability to use pre-operative standing lumbar extension radiographs and supine MRI to predict the amount of radiographic sagittal deformity correction.

Although adequate radiographic correction in the sagittal plane was achieved immediately post-operatively, this study revealed a subtle loss of the corrected LL over time in most patients (87%). After a mean of 29 months of follow-up, approximately 4^o^ of the LL achieved initially was lost. The clinical implication of this radiographic finding is unknown. Several studies have reported a similar loss of LL over time following lumbar instrumented fusions. Dimar et al. observed about a 3^o^ loss of LL following instrumented posterolateral fusion from L3 to S1 over a minimum of 6 months of follow-up [12]. Even with anterior column support with the use of interbody cages, other authors have reported similar findings at 2-year follow-up with a mean loss of LL of 3.4^o^ [26]. These numbers are in line with our findings. Importantly, there was no reported effect on clinical outcome in either of these studies.

An interesting finding in our study was that while pre-operative standing lumbar extension radiographs were better at predicting a post-operative PI-LL mismatch of 10^o^ or less immediately post-operatively, measuring the LL on pre-operative MRI provided a better predictor of maintenance of that success for over 2 years of follow-up. This is perhaps because lumbar extension radiographs are looking at a dynamic position of the lumbar spine and that those patients who were able to achieve a PI-LL mismatch within 10^o^ on these films have a more flexible spine than those who could only achieve that mismatch on their MRI but not on their lumbar extension radiographs. This additional flexibility could perhaps be responsible for the loss of LL over time in this group. Thus, we may be able to predict the radiographic success of sagittal plane correction with our technique by assessing pre-operative spinal flexibility. This flexibility was estimated by measuring the changes in LL between various routine pre-operative images. These easy-to-perform measurements can be very helpful when determining if a longer fusion construct is required to correct sagittal plan deformity or if similar correction can be achieved by performing a lesser, more targeted fusion of only the symptomatic levels. Certainly, this concept of quantifying spinal flexibility must be further validated using larger sample sizes and investigated to determine if it can also be applied to spinal pathologies other than DS.

In our experience, the radicular symptoms present in these patients commonly originate from foraminal stenosis at the level of the lumbosacral fractional curve, which is typically caudal to the main DS curve. These radicular symptoms predominantly occur from L5 and/or S1 nerve root compression, as observed in 80% (54 of 68) of our patients. Previous studies described this relationship between the side and pattern of radiculopathy and the side of the curve concavity. Simmons et al. described that patients with radiculopathy and scoliosis presented with symptoms that originated from lumbar curve concavities [27]. Specifically, 7 of 9 patients (77.8%) experienced L5 and S1 radiculopathy that originated on the concave side of the lumbosacral fractional curve. Recently, Liu et al. identified symptomatic radiculopathy in adults with DS using selective nerve root injections [4]. The same authors found nerve root compression at the lumbar structural curve concavity of L3 and/or L4 in 15 of 20 patients, and nerve root compression at L5 and/or S1 on the lumbosacral fractional curve concavity side in 12 of 16 patients.

In this cohort of DS patients, symptomatic foraminal stenosis within the lumbosacral fractional curve concavity was addressed with an instrumented fusion for fear of inadequacy of decompression alone. This is because foraminal compression of the nerve root in this setting often occurs in the cranial-caudal direction in addition to the more usual anterior-posterior direction. The former is often referred to as “pedicular kinking.” Even though the anterior-posterior stenosis can typically be addressed with a decompression-only procedure, cranial-caudal stenosis is much more difficult to address without distracting the foramen with either transpedicular instrumentation or the application of an interbody cage [28]. This pedicular kinking occurs in the setting of advanced disc degeneration and is often exacerbated with weight-bearing activities. Hasegawa et al. described critical dimensions of foraminal stenosis to be ≤ 4 mm of posterior disc height and ≤ 15 mm of foraminal height. In our patient population, the mean foraminal height within the fractional curve concavity was 15 mm. This decreased foraminal height is in line with Hasegawa’s study [29]. In addition to providing immediate stability, transpedicular instrumentation can aid in indirect decompression of the foramen by the reduction of pedicular kinking following segmental pedicular distraction using the instrumentation. Infusa and colleagues were able to increase the foraminal dimensions by 39.6% at L5-S1 and by 22.6% at L4–5 with only 6 mm of rod distraction [30].

There are several limitations to this study: (1) Our cohort consisted of a relatively small number of patients and lacked long-term follow-up. (2) We did not obtain subjective or objective clinical outcomes for the majority of these patients including range of motion, symptom relief, or pain. Although we describe significant improvements in radiographic measurements, no direct correlations with clinical outcomes can be made. (3) Our results can only be applied to a very specific cohort of DS patients—those who underwent a targeted decompression and fusion of only their neurologically symptomatic levels. This is a “selected” cohort comprised of patients with “moderate” scoliosis with a Cobb angle less than 40°, coronal shift less than 2 cm, and a SVA less than 10 cm. We did not study a control/comparative group, as the goal of the study was to specifically evaluate the radiographic outcomes of patients treated with this targeted strategy. Certainly, there were many additional DS patients treated at our institution during this time that necessitated regional or global fusions because either their deformities were of a greater severity or they experienced symptoms outside of the fractional curve. This diversity in DS patients further underscores the need to “personalize” management options in these patients in order to optimize outcomes. For the purposes of this study, we did not take into account the patients age, specific symptomatology, or etiology of the disease. We recognize that the progression of degenerative scoliosis is multifactorial in which age and etiology play significant roles, and the loss of lordosis and subsequent post-operative improvement could directly related to those factors. As such, we will focus our prospective research on evaluating these patients in the context of their age, specific symptoms, and etiology of presentation.

## Conclusion

This is the first study to demonstrate that when DS patients have sagittal deformities that are flexible on pre-operative imaging, a targeted instrumented arthrodesis and decompression of only the neurologically symptomatic levels can achieve a PI-LL mismatch to within 10^o^. This result was maintained through 29.2 months of follow-up. We also demonstrate for the first time that the magnitude of pre-operative spinal flexibility is correlated with the amount of post-operative radiographic correction of sagittal plane parameters after fusion, giving the surgeon a simple tool to predict who this targeted surgical technique may be the most beneficial for. This more targeted surgical strategy could prevent some of the intraoperative and post-operative complications often seen with longer instrumented fusions, osteotomies, or interbody fusions.

While there are many DS patients for whom this more targeted treatment approach would not work, our preliminary evidence indicates that this approach appears to work well in carefully selected patients (Cobb angle of less than 40^o^ and SVA less than 10 cm positive) whose neurogenic symptoms originate predominantly from a fractional lumbosacral curve. In our practice, longer regional or global fusions for sagittal and coronal balance correction are often indicated in the presence of progressive collapse without a single level identified as a pain source or in the case of more severe curves. It is our practice that patients with a particularly large DS curve are specifically counseled about the possibility of future adjacent segment degeneration or that all of their symptoms may not be addressed by a targeted fractional curve fusion alone. Further studies to identify clinical outcomes of DS patient treated with this targeted strategy will be important to assess for any adverse outcomes secondary to the loss of LL over time. It is important to note that these are preliminary findings and that further comparative and prospective studies are necessary to better understand these concepts as well as to replicate our findings.

## Abbreviations

AUC: Area under the curve
CI: Confidence intervals
DS: Degenerative scoliosis
HRQOL: Health-Related Quality Of Life Outcomes
IRB: Institutional Review Board
LL: Lumbar lordosis
MRI: Magnetic resonance imaging
OR: Odds ratio
PI: Pelvic incidence
PJK: Proximal junctional kyphosis
PT: Pelvic tilt
ROC: Receiver operating characteristic
SS: Sacral slope
